# Application of testicular organ culture system for the evaluation of spermatogenesis impairment

**DOI:** 10.1038/s41598-024-71561-6

**Published:** 2024-09-16

**Authors:** Hideaki Nakagiri, Takehiko Ogawa, Naohiro Ikeda, Shimpei Terasaka, Yuko Nukada, Masaaki Miyazawa

**Affiliations:** 1 Safety Science Research Laboratories, Kao Corporation, Haga, Tochigi 321-3497 Japan; 2 Department of Regenerative Medicine, Graduate School of Medicine, Yokohama City University, 3-9 Fukuura, Kanazawa-Ku, Yokohama, Kanagawa 236-0004 Japan

**Keywords:** Testicular toxicity, In vitro spermatogenesis, Testicular organ culture system, PMDS, PC method, Reproductive disorders, Infertility

## Abstract

Recently, it was reported that a testicular organ culture system (TOCS) using polydimethylsiloxane (PDMS) chips with excellent oxygen permeability and biocompatibility, called the PDMS-chip ceiling (PC) method, enables improved spermatogenesis efficiency. We investigated whether this PC method is useful for detecting impaired spermatogenesis caused by busulfan (Bu), a typical testicular toxicant. In this study, testicular tissue fragments from Acro3-EGFP mice, which express the green fluorescent protein (GFP) and reflect the progression of spermatogenesis, were subjected to the PC method. When treated with Bu, cultured tissues shrank in volume, and their GFP-expressing area decreased or disappeared. Histological examination confirmed the regression of spermatogenesis. In addition, immunohistochemical examination revealed that spermatogonia, including spermatogonial stem cells (SSCs), were the primary targets of Bu toxicity. Time-course analysis demonstrated that the recovery of spermatogenesis, dependent on Bu concentration, correlated closely with the severity of damage to these target cells. These results suggest that the PC method is a useful approach for detecting spermatogenesis impairment accurately through faithful recapitulation of spermatogenesis in vivo.

## Introduction

Infertility has become a worldwide social problem, and studies have shown that the male side causes contribute to approximately 50% of infertility cases, with spermatogenesis impairment being the leading issue^1,2^. The causes of impaired spermatogenesis can be broadly divided into congenital and acquired factors^3^. Acquired factors include lifestyle factors, such as excessive and habitual alcohol consumption and smoking, as well as diseases, such as diabetes mellitus and exposure to pollutants. However, most of those impairment mechanisms remain unresolved.

Spermatogenesis begins with the continuous proliferation of spermatogonia, followed by meiotic recombination in spermatocytes, dynamic morphological changes, and the transformation of nucleoproteins in spermatids. These processes are highly complex and regulated^4–6^. It is important to develop a system that can reproduce spermatogenesis in vitro to elucidate the details of the molecular and cellular mechanisms of spermatogenesis and understand the pathogenesis of its impairment.

Sato et al. successfully reproduced mouse spermatogenesis in vitro using the agarose gel as a stand for placing testis tissues (AG method), a testicular organ culture system (TOCS) based on the classical air–liquid interface method^7^. Studies investigated hormones and chemicals, revealing some as essential or promotional for spermatogenesis^8,9^. In the TOCS, it is possible to control the concentration of various substances in the medium and the exposure duration for each cultured tissue. It also allows for continuous and in-depth observation of the cultured tissues over an extended period of time. When studying the effects of particular agents, toxicants in particular, on spermatogenesis, these are advantages.

There have been several reports that applied TOCS for testicular toxicity evaluation^10–13^. These reports used a gas–liquid interphase method in which tissue fragments were cultured on agarose gels or culture insert membranes. Tissues cultured in such ways usually showed extensive degeneration and necrosis in their central region, which would be difficult to distinguish from damages in toxicological studies^14^. Therefore, it is necessary to improve the heterogeneity of spermatogenesis in cultured tissues for a more precise toxicity evaluation. To achieve this, a new TOCS was developed using a microfluidic system made of polydimethylsiloxane (PDMS), a material with high biocompatibility and excellent oxygen permeability^15–19^. Tissues cultured in microfluidic devices made of PDMS were shown to induce spermatogenesis more extensively than those in AG methods and maintain spermatogenesis faithfully over a longer period. However, this microfluidic device requires an extremely complex and precise design, rendering its production time-consuming and labor-intensive. Then, another method, the PDMS-chip ceiling (PC) method, which uses PDMS chips, appeared as an improved version of the culture method. It is simple yet has the advantages of a microfluidic device, including improved spermatogenesis efficiency without tissue degeneration in the center of the tissue^20,21^. Most importantly, the manufacturing of PC chips is easier than the labor required to manufacture microfluidic devices.

This study used the PC method to verify whether it could provide useful information for detecting spermatogenesis impairments. Busulfan (Bu), an alkylating agent, was used as a representative testicular toxicant to damage spermatogenesis^22,23^. Bu disrupts the DNA structure of spermatogonia, including spermatogonial stem cells (SSCs), preventing their proliferation and differentiation and inducing apoptosis, thereby leading to impaired spermatogenesis^23,24^. It is reported in animal experiments that low-dose Bu exposure impairs spermatogenesis but allows for regeneration; however, at high-dose exposures, germ cells are completely depleted, resulting in the irreversible devastation of spermatogenesis. Although there have been few reports of culture experiments demonstrating findings similar to those observed in animal studies, we successfully showed, using the PC method, that such impairment and recovery of spermatogenesis can occur under culture conditions. One of the key advantages of culture experiments is the ability to observe the same tissue over time repeatedly. In this regard, the green fluorescent protein (GFP) expression index, using Acro3-EGFP mice^25,26^, which expresses GFP during spermatogenesis, has been useful and validated through histological evaluation. In this study, to verify the usefulness of our culture system for toxicity evaluation, we conducted a quantitative immunohistochemical analysis of the target germ cells, specifically spermatogonia and SSCs. We linked these findings to the temporal changes in tissue GFP expression and histological evaluation following treatment with toxic substances.

## Results

### In vitro spermatogenesis using the PC method

The PC chips were manufactured in this study based on previous reports^21,27^ (Supplementary Fig. S1). The newly manufactured chips have a circular depression with a diameter of 8 mm and a depth of 160 µm. Because the materials used for this chip differed from those previously reported, testicular tissue fragments were first cultured using this chip to evaluate the induction and maintenance of spermatogenesis.

**Fig. 1:**
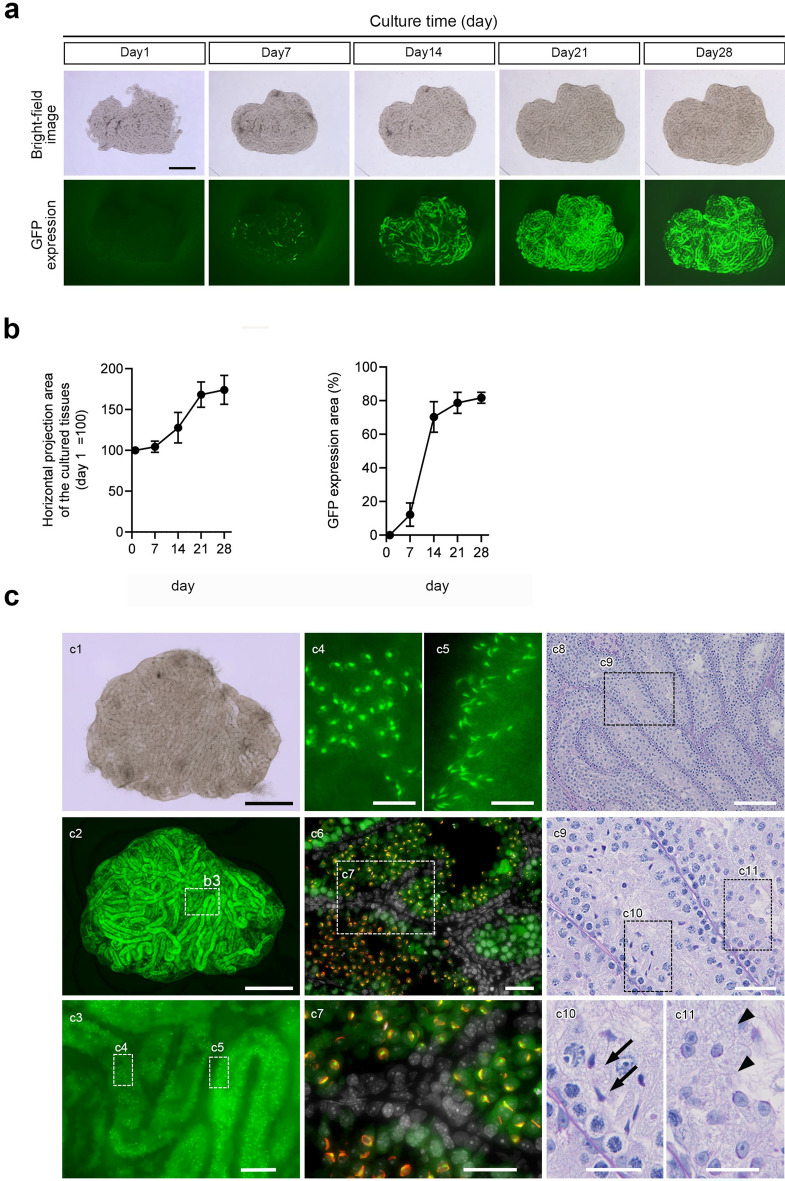
In vitro spermatogenesis using the PC method. (**a**) The horizontal projected and GFP expression areas of testis fragments (5–7 dpp) from Acro3-EGFP mice cultured for 4 weeks with PC chips are chronologically demonstrated. (**b**) The horizontal projection area changed the rate of the tissue, and the GFP expression area ratio (%) to the cultured tissue area was quantified. Values represent mean (SD); N = 6 tissues in each group. (**c**) An inverted microscopic image of tissue from a 6 dpp mouse cultured for 65 days, fixed in 4% PFA, and observed under an inverted fluorescence microscope. (**c1**) Bright-field and (**c2**) GFP expression. Testis tissues cultured on the PC chips showed widespread GFP expression. (**c3**) Enlarged image of the dashed line rectangle in (**c2**). (**c4**, **c5**) Enlarged image of the dashed line rectangle in (**c3**). GFP was concentrated in an acrosomal shape that represented round and elongating spermatids. (**c6**) Immunohistochemical staining of cultured tissue. GFP, green; PNA, red; DAPI, white. **(c7**) Enlarged image of the dashed line rectangle in (**c6**). GFP-CAP shape co-localized with PNA lectin was observed using IHC. (**c8**). PAS staining of cultured tissues for 53 days. (**c9**) Enlarged image of the dashed line rectangle in (**c8**). (**c10**, **c11**) Enlarged image of the dashed line rectangle in (**c9**). Elongating (**c10**; arrowhead) and round (**c11**; arrow) spermatids were observed. Scale bars: 1 mm (**c1**, **c2**), 100 µm (**c3**), 20 µm (**c4**, **c5**), 40 µm (**c6**, **c7**), 100 µm (**c8**), 40 µm (**c9**), and 20 µm (**c10**, **c11**).

**Fig. 2:**
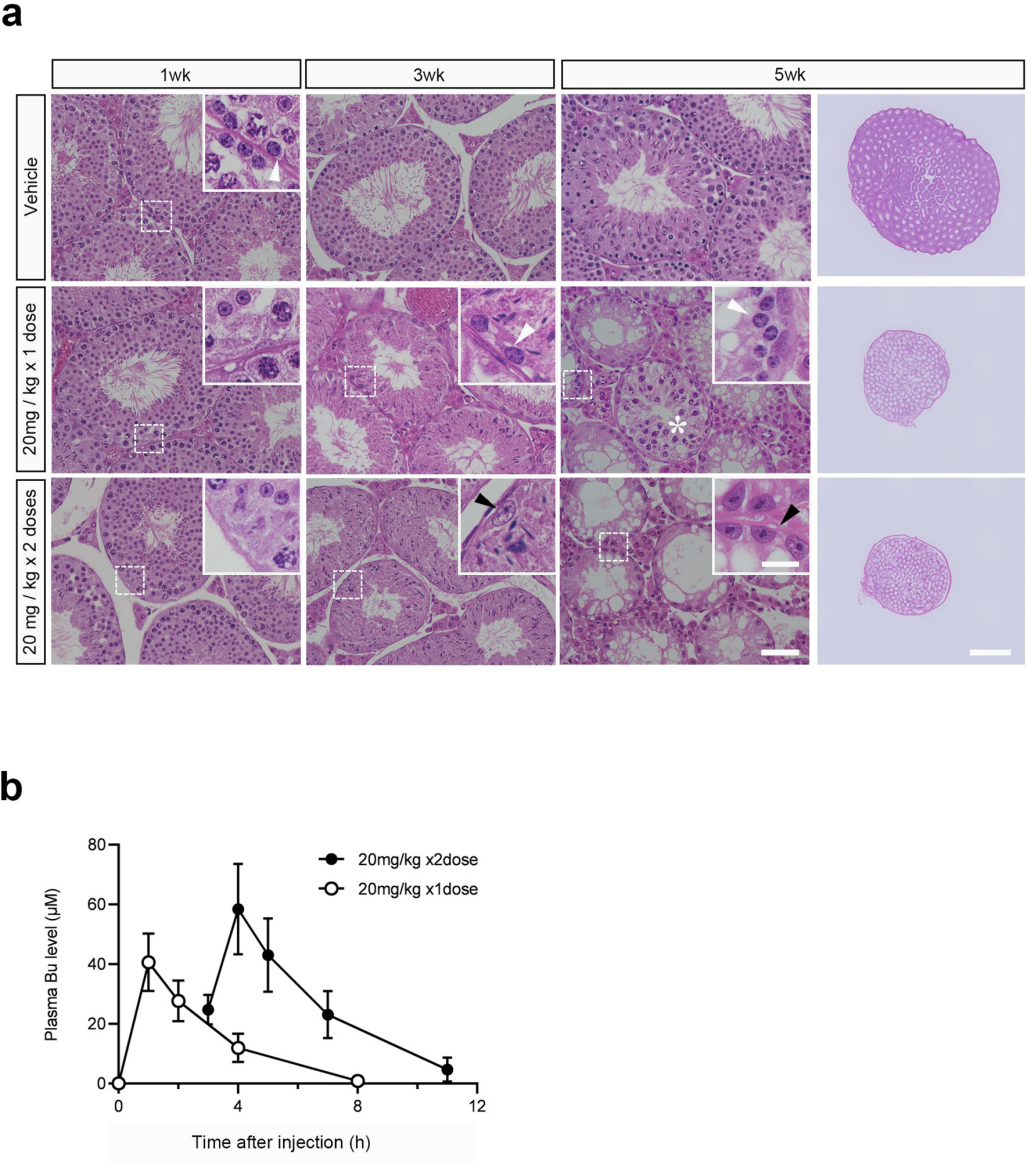
Impaired spermatogenesis and toxicokinetics after Bu treatment. (**a**) Histological examination after Bu treatment in vivo. Mice (C57BL/6, 8 weeks old, N = 5 mice) were intraperitoneally administered Bu at a dose of 20 mg/kg once or twice with a 3 h interval between injections. Hematoxylin and eosin staining of the testes after Bu injection is shown chronologically (after 1, 3, and 5 weeks of treatment). An equal amount of DMSO was injected as a vehicle (control). The dashed rectangular areas are enlarged in the insets. White arrowheads indicate spermatogonia on the basement membrane. Black arrowheads indicate Sertoli cells. The asterisk shows a tubule containing spermatocytes. Scale bars: upper row, 1 mm; bottom row (inset), 50 μm and 20 µm. (**b**) Toxicokinetics of Bu after intraperitoneal injection. Mice (C57BL/6, 8 weeks old, N = 5 mice) were intraperitoneally administered Bu at a dose of 20 mg/kg once or twice at 3 h intervals. Blood samples were collected at each time point. In the case of two Bu injections, blood was collected immediately before the second injection. The plasma concentration of Bu was measured by LC–MS/MS. C_max: 2dose_ = 58 ± 15 µM, C_max: 1dose_ = 41 ± 10 µM. Values represent mean ± SD.

**Fig. 3:**
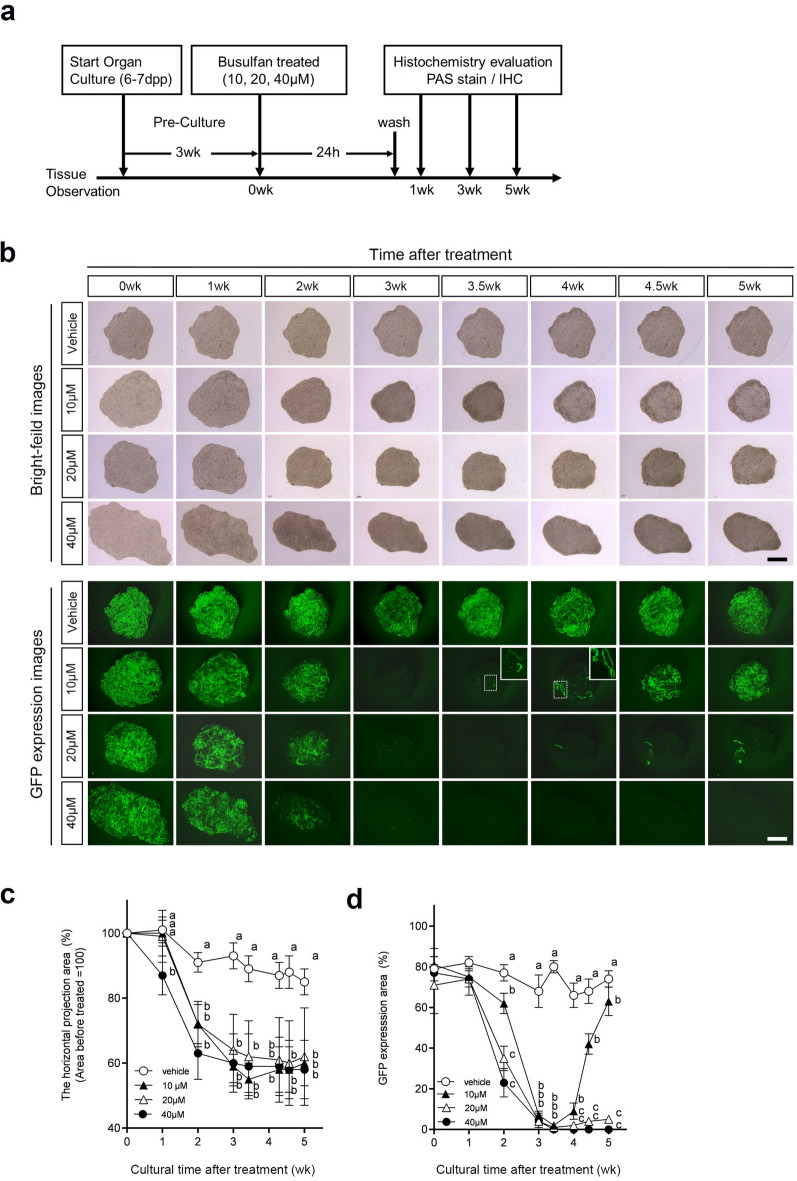
Observation of cultured testis fragments after Bu treatment. (**a**) Scheme of the experimental procedure. Testes from 6 to 7 dpp Acro3-EGFP mice were subjected to organ culture using the PC method. After 3 weeks of pre-culture, when GFP was expressed throughout the tissue fragments and had stabilized, cultured tissue fragments were treated with 10, 20, and 40 µM Bu for 24 h. Cultured tissue fragments were histologically evaluated after 1, 3, and 5 weeks of Bu treatment. Cultured tissue fragments were observed under a stereomicroscope over time before Bu treatment. (**b**) The horizontal projection and GFP expression areas of testicular tissue fragments cultured for 5 weeks after treatment with Bu (10, 20, and 40 μM) are shown chronologically. Both areas of tissues treated with each concentration were obtained from the same tissue. The dashed rectangular areas are enlarged in the insets. Scale bars: 1 mm. (**c**) Quantification of changes in the horizontal projection area of the cultured tissue after Bu treatment. Different letters (a, b) indicate significant differences between the groups (*P* < 0.05). Values represent mean ± SD; N = 6 tissues in each group. (**d**) The ratio of the GFP-expressing area to the horizontal projected area of cultured tissue after Bu treatment was quantified. Different letters (a, b, c) indicate significant differences between the groups (*P* < 0.05). Values represent mean ± SD; N = 6 tissues in each group. *Bu* busulfan, *GFP* green fluorescent protein.

**Fig. 4:**
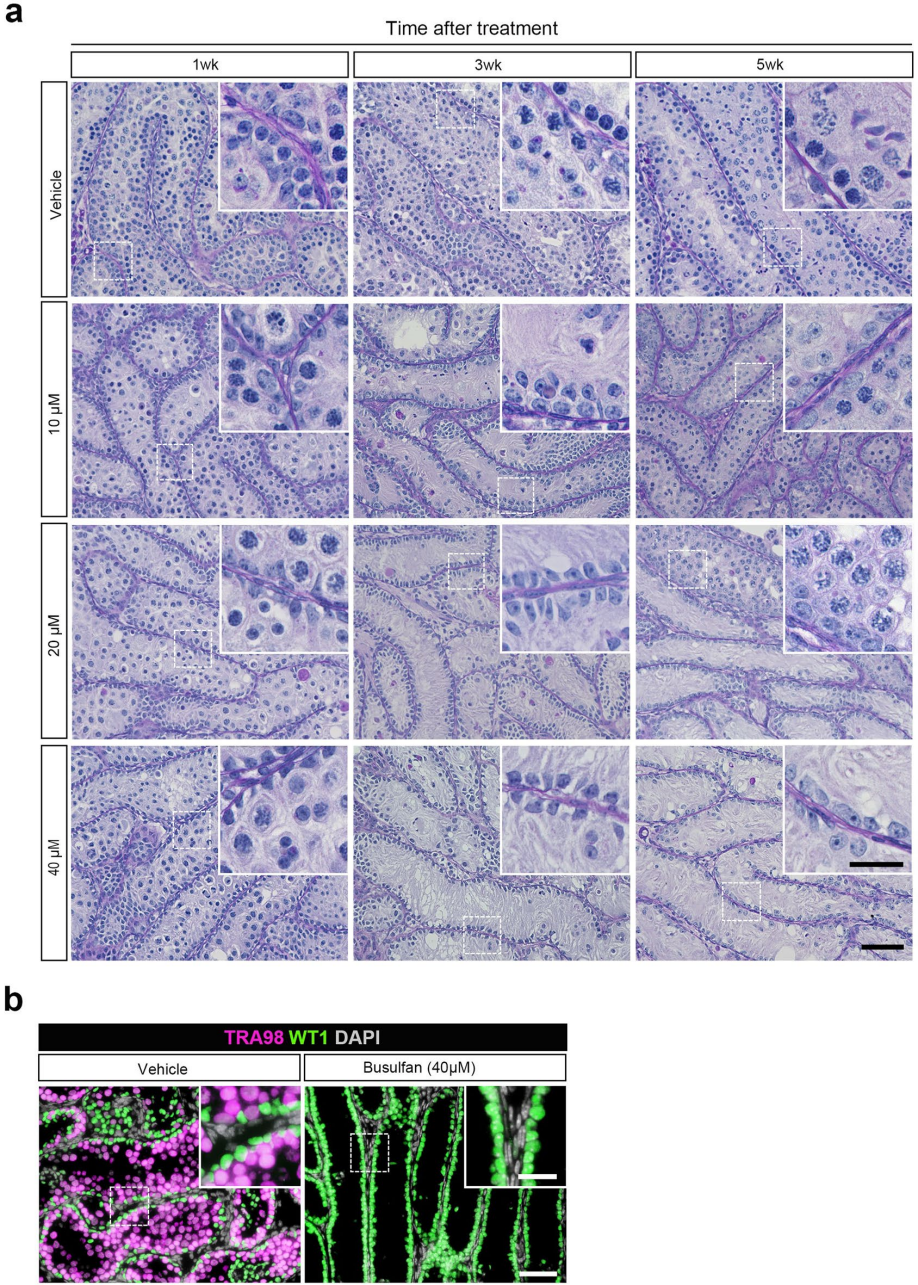
Histological examination of cultured testis fragments after Bu treatment. (**a**) PAS staining of cultured tissues at each time point after treatment with different Bu concentrations is shown. The dashed rectangular areas are enlarged in the insets. Scale bars: 50 µm and 20 µm (inset). (**b**) IHC of WT1 is shown in the cultured tissue 5 weeks following Bu treatment. WT1 (+) cells, green; TRA98, magenta; DAPI, white. Dashed rectangular areas are enlarged in the insets. Scale bars: 50 µm and 20 µm (inset).

**Fig. 5:**
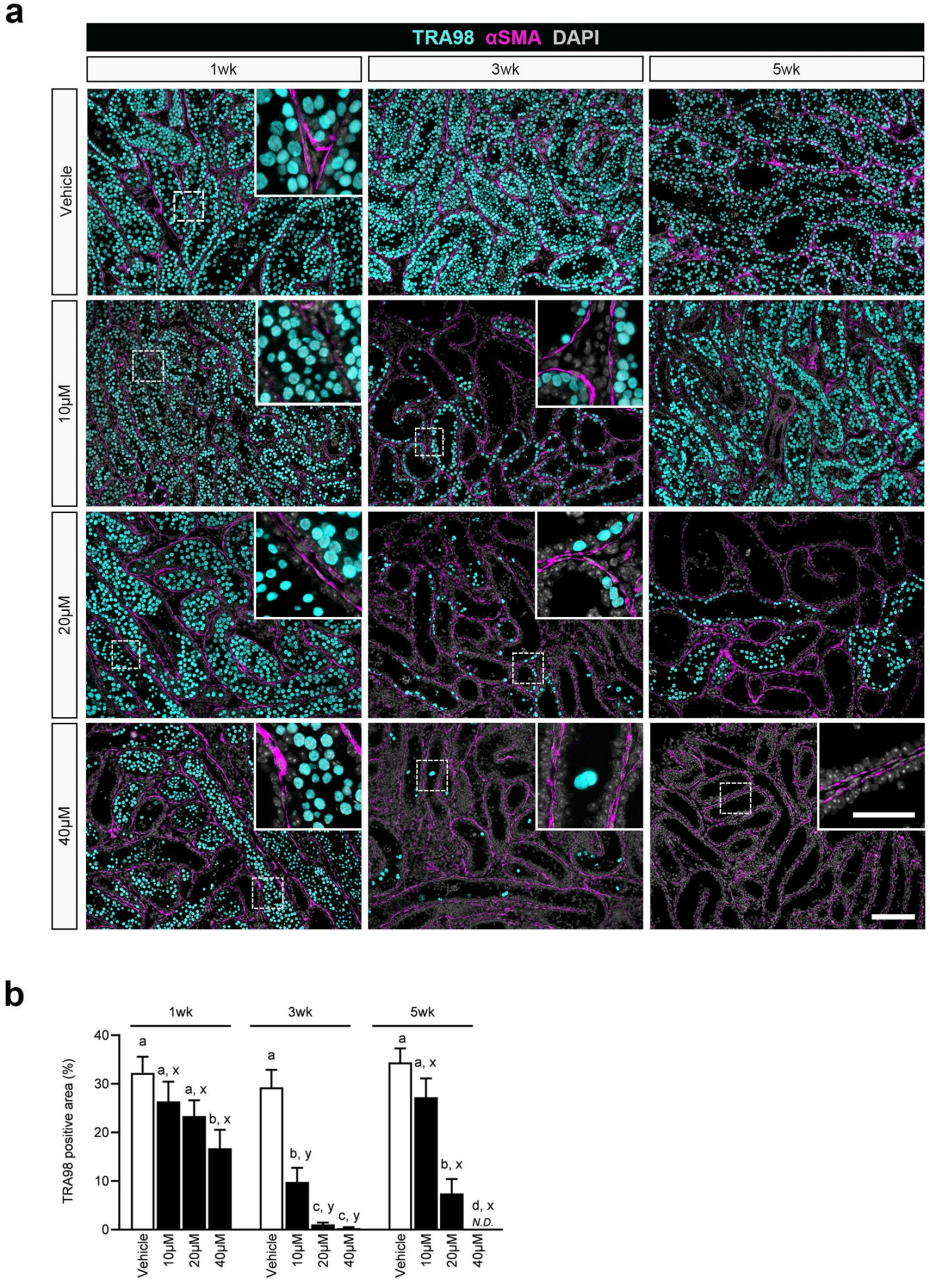
Effect of Bu treatment on germ cells in cultured testis. (**a**) IHC of TRA98 is shown in the cultured tissue after Bu treatment at 1, 3, and 5 weeks. TRA98 (+) cells, cyan; α-SMA, magenta; DAPI, white. Dashed rectangular areas are enlarged in the insets. (**b**) The TRA98 (+) area per tissue section area after Bu treatment was quantified based on IHC images. Positive areas were calculated using a hybrid cell count application software (BZ-H4A, KEYENCE, Osaka, Japan). The dashed rectangular areas are enlarged in the insets. Different letters (a–d) indicate significant differences between groups at each time point (*P* < 0.05). *N.D.*: not detected. Different letters (x, y, z) indicate significant differences between times (1, 3, and 5 weeks) for the same treatment (*P* < 0.05). Values represent mean ± SD; N = 4 tissues in each group. Scale bars: 100 µm and 40 µm (inset).

**Fig. 6:**
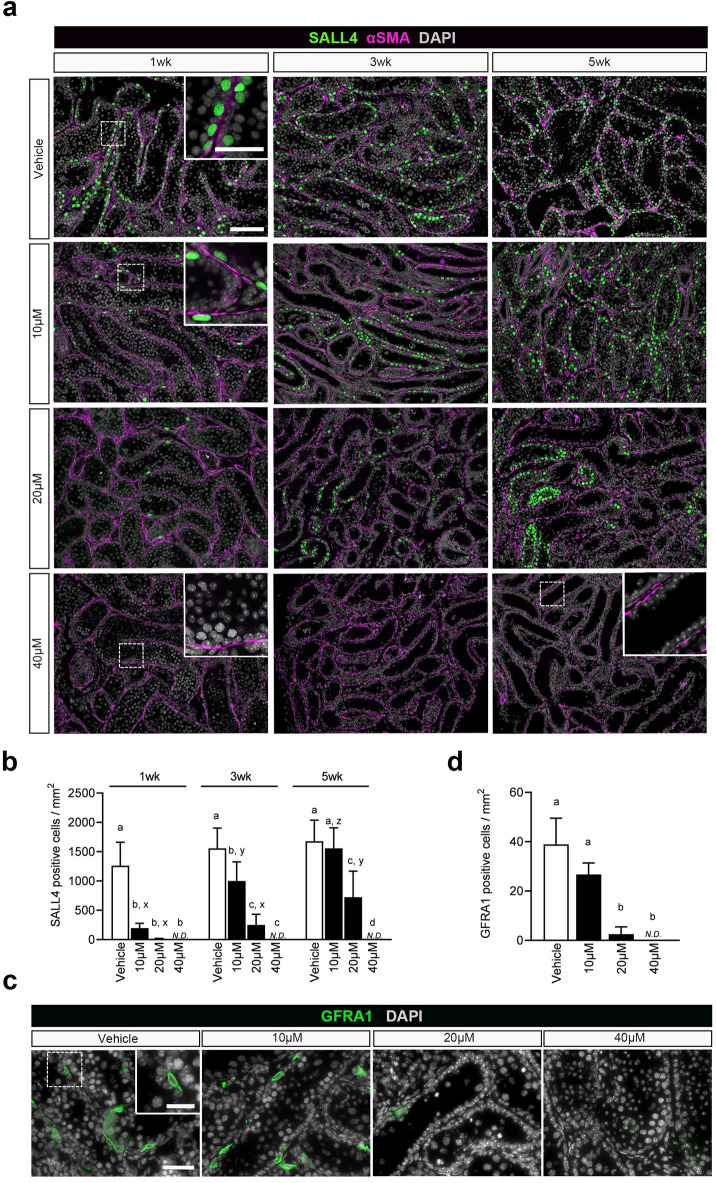
Evaluation of Bu-treated cells in the cultured testis. (**a**) IHC of SALL4 is shown in the cultured tissue after Bu treatment at 1, 3, and 5 weeks. SALL4 (+) cells, green; α-SMA, magenta; DAPI, white. Dashed rectangular areas are enlarged in the insets. (**b**) The number of SALL4 (+) cells per tissue section area after Bu treatment was counted based on the IHC images. Different letters (a, b, c, d) indicate significant differences between groups at each time point (*P* < 0.05). Different letters (x, y, z) indicate significant differences between times (1, 3, and 5 weeks) for the same treatment (*P* < 0.05). *N.D.*: not detected. Values represent mean ± SD; N = 4 tissues in each group. Scale bars: 100 µm and 40 µm (inset). (**c**) IHC image of GFRA1 in cultured tissues after treatment for 1 week. GFRA1 (+) cells, green; DAPI, white. Dashed rectangular areas are enlarged in the insets. (**d**) The number of GFRA1 (+) cells per tissue section after Bu treatment was counted based on the IHC images. Values represent mean ± SD; N = 4 tissues per group. *N.D.*: not detected. Different letters (a, b) indicate significant differences between the groups (*P* < 0.05). The dashed rectangular areas are enlarged in the insets. Scale bars: 40 µm and 20 µm (inset).

Testis tissue fragments from Acro3-EGFP mice at 5–7 days post-partum (dpp) were subjected to culture experiments. Bright-field images of the cultured tissue and GFP expression images were observed once a week after the first day of culture (Fig. 1a). The tissue fragment area steadily increased after the culture started and reached a steady size on day 21. GFP expression was partially observed along the seminiferous tubules on day 7 of culture, spread throughout seminiferous tubules in the tissue on day 21, and remained stable thereafter. The horizontal projected area of the cultured tissue and GFP expression area were quantified (Fig. 1b). The relative tissue area spread approximately 1.7-fold after 21 days of culture, compared with day 1 of culture, and then stabilized. In contrast, the GFP expression area spread rapidly from day 14 of culture, reaching approximately 80% of the horizontal projection area of the cultured tissue after 21 days of culture and then stabilized. In the 65-day cultured tissues, GFP expression was observed throughout the tissue (Fig. 1c-1,c-2), and high magnification showed that GFP accumulated into acrosomes whose shape represented round and elongated spermatids (Fig. 1c-3–5). Immunohistochemistry (IHC) with peanut agglutinin (PNA) lectin, a marker of the acrosome, confirmed that those GFP co-localized with PNA lectin (Fig. 1c-6,c-7).

In contrast, GFP expression was not observed in cells near the basement membrane. They were pre-meiotic spermatogonia and Sertoli cells (Fig. 1c-6,c-7). Furthermore, histological examination using periodic acid-Schiff (PAS) staining revealed round and elongated spermatid cells in the seminiferous tubules of the cultured tissues (Fig. 1c-8–11). Most of the seminiferous tubules, including those located in the tissue center, had spermatocytes or round spermatocytes (Supplementary Fig. S2). Taken together, the newly manufactured PC chip, which was made of a different material described in the “Methods” than that used in the previous report, successfully reproduced spermatogenesis, as reported previously. Hence, we decided to use this chip in subsequent studies.

### Spermatogenesis impairment in vivo and plasma Bu levels

A previous study reported a significant reduction or total elimination of germ cells in mice following one or two intraperitoneal injections of 20 mg/kg Bu at intervals of 3 h^28,29^. We decided to use this experimental scheme to measure the plasma concentrations of Bu to determine the suitable Bu concentration in culture media of in vitro studies. After 1 week of intraperitoneal Bu treatment, histological examination showed that many germ cells remained in the seminiferous tubules in the testes, regardless of the dose. However, close examination revealed that the number of spermatogonia on the basement membrane was reduced (Fig. 2a). After 3 weeks of treatment, germ cells in the seminiferous tubules were considerably reduced in both the Bu single- and double-dose groups, and the remaining germ cells were mostly elongated spermatids. In the single-dose Bu group, there were a few tubules containing a few spermatogonia remaining on the basement membrane (Fig. 2a); while in the double-dose Bu group, almost all spermatogonia disappeared, and only Sertoli cells were observed on the membrane (Fig. 2a).

After 5 weeks of treatment with a single dose of Bu, spermatocytes and spermatogonia were observed in some seminiferous tubules, suggesting regeneration of spermatogenesis (Fig. 2a). In contrast, germ cells remained depleted in the double-dose Bu group. Atrophy of seminiferous tubules and testes was observed in both groups (Fig. 2a). The plasma Bu concentration in the single dose group showed a C_max_ value of approximately 40 µM at 1 h after administration (Fig. 2b). In contrast, in the double dose group, the C_max_ value was approximately 60 µM at 1 h after the second administration (Fig. 2b). Based on these results, we decided to set 10, 20, and 40 μM as the Bu concentration in the media, expecting to observe severe damage and recovery of spermatogenesis under TOCS condition.

### Stereomicroscopic observation of cultured tissue after Bu treatment

Figure 3a illustrates the experimental scheme. Testicular tissue fragments from mice aged 5- to 7-dpp were cultured for 3 weeks, during which GFP expression became widespread throughout the tissue and stabilized. Bu was then applied to the tissue by adding it to the culture medium. After a 24-h treatment with Bu, the culture medium was changed thrice every 3 h to remove Bu. Subsequently, the cultured tissues were observed weekly and histologically evaluated at 1, 3, and 5 weeks. Bright-field and GFP images of cultured tissues were taken over time for 5 weeks (Fig. 3b). Although there was no change in the horizontal projection area of the tissue in the bright-field images of the vehicle group, a clear decrease (i.e., tissue atrophy) was observed after the second week in the Bu-treated group. The Bu-treated group showed a significantly greater reduction than the vehicle-treated group at all concentrations (Fig. 3c). In the 40 µM Bu-treated group, specifically, the horizontal projection area of the cultured tissue was significantly and markedly reduced compared to that in the vehicle group after 1 week of treatment (Fig. 3b,c). The GFP expression area (%) remained almost constant for 5 weeks in the vehicle group, whereas GFP expression in the Bu-treated group almost disappeared by week 3 at all concentrations (Fig. 3b). Furthermore, the GFP expression area (%) showed a significant concentration-dependent decrease compared to the vehicle group after 2 weeks of Bu treatment (Fig. 3b,d). In particular, the tissues treated with 40 µM Bu lost GFP expression completely after 3.5 weeks (Fig. 3b,d).

However, GFP expression along the tubules was observed in tissues treated with 10 and 20 µM Bu after 3.5 and 4 weeks of treatment (Fig. 3b, inset). In particular, the GFP expression area (%) in the 10 µM Bu-treated group recovered rapidly to the same level as that in the vehicle group after 5 weeks of treatment. Conversely, in the 20 μM Bu-treated group, the regeneration was limited with only a slight increasing trend in GFP expression area (%) after 4 weeks onward.

### Histological evaluation after Bu treatment

Histological evaluation was performed to confirm that changes in GFP expression in the cultured tissues adequately reflected the state of spermatogenesis after Bu treatment. PAS staining was performed on the tissues at each treatment concentration chronologically (Fig. 4a). At 1, 3, and 5 weeks, round or elongated spermatids were observed in the seminiferous tubules of the cultured tissues in the vehicle group (Fig. 4a; inset). Many germ cells disappeared after 3 weeks of Bu treatment at any concentrations. However, spermatogonia were observed on the basement membrane in the tissues treated with 10 µM Bu (Fig. 4a; inset). After 5 weeks of treatment, a substantial number of germ cells was again observed in most of the seminiferous tubules in the 10 µM Bu-treated group (Fig. 4a; inset). In contrast, in the 20 µM Bu-treated group, germ cells were observed only in some of the tubules of the cultured tissue (Fig. 4a; inset), whereas, in the 40 µM Bu-treated group, no germ cells were observed, leaving only Sertoli cells, which are WT1 positive, in the seminiferous tubules (Fig. 4b; inset). The changes in these histological findings were consistent with changes in GFP expression (Fig. 3b).

### Effect of Bu treatment on germ cell number in cultured testis

To better understand Bu-induced impairment of spermatogenesis, additional IHC was performed to quantify changes in germ cell numbers after Bu treatment. TRA98 was used as a pan-germ cell marker. The number of positive cells at each time point, 1, 3, and 5 weeks, was evaluated (Fig. 5a). As the IHC images showed many overlapping TRA98-positive nuclei, which were difficult to count, TRA98-positive regions were measured and quantified using the BZ-X Analyzer software described in the “Methods” (Fig. 5b). At 1 week after Bu treatment, many TRA98-positive cells were observed in every group, and the positive area became smaller as Bu-concentration increased. In particular, the 40 µM Bu-treated group showed significantly smaller TRA98-positive areas than those in the vehicle and other Bu-treated groups (Fig. 5a,b). At 3 weeks, the TRA98-positive area was significantly smaller and decreased in all Bu treatments compared to 1 week after treatment. The TRA98-positive area in the vehicle group remained almost unchanged, whereas a significant decrease was observed in the Bu-treated group (Fig. 5a,b). At 5 weeks, the TRA98-positive area became significantly larger in the 10 and 20 µM Bu-treated groups than after 3 weeks of treatment, reflecting the recovery of spermatogenesis. In particular, the TRA98-positive area in the 10 µM Bu-treated groups was not significantly different from that of the vehicle group and spread throughout the tissue (Fig. 5a,b). On the other hand, no TRA98-positive area was detected in the 40 µM Bu-treated groups (Fig. 5a,b).

### Evaluation of Bu target cells in the cultured testis

As the target cells of Bu are considered to be spermatogonia^22,23^, changes in the number of spermatogonia were measured immunohistochemically using SALL4 as the marker of spermatogonia^30^ (Fig. 6a). At 1 week of Bu treatment, the number of SALL4-positive cells decreased significantly in every Bu-treated group compared to that in the vehicle group. No SALL4-positive cells were observed in the 40 µM Bu-treated group (Fig. 6a,b). At 3 weeks of Bu treatment, the 10 µM Bu-treated group showed significantly higher numbers of SALL4-positive cells than those in the first week after treatment, reflecting the recovering proliferation of spermatogonia. The 20 µM Bu-treated group also showed a trend of recovery (Fig. 6a,b). At 5 weeks of Bu treatment, the number of SALL4-positive cells increased significantly and considerably in the 10 and 20 µM Bu-treated groups than at 3 weeks of treatment. In particular, the number of positive cells in the 10 µM Bu-treated tissue increased to a level comparable to that in the vehicle group (Fig. 6a,b). In the 40 µM Bu-treated group, SALL4-positive cells disappeared from week 1 and did not recover even at week 5. These results indicate that Bu toxicity strongly affects SALL4-positive cells, namely spermatogonia, among germ cells and is consistent with the results of previously reported in vivo studies^22,23^.

Then, we examined the effect of Bu on SSCs among all spermatogonia using GFRA1 as a marker of SSCs^31,32^. As the most significant decrease in spermatogonia (SALL4-positive cells) was observed as early as 1 week after Bu treatment, cultured tissue from the same time period was evaluated (Fig. 6c,d). The number of GFRA1-positive cells in the cultured tissue was reduced in the 10 μM Bu-treated group but was not significantly different from that in the vehicle group. In the 20 µM Bu-treated group, the number of GFRA1-positive cells significantly decreased, and only a few residual cells were observed. No GFRA1-positive cells were observed in the 40 µM Bu-treated group. Hence, the number of GFRA1-positive cells in the Bu-treated groups decreased in a Bu concentration-dependent manner, as in the case of SALL4-positive cells. However, in the 10 µM Bu-treated group, 1 week after the Bu-treatment, GFRA1-positive cells remained at about 70% of control, while SALL-4 positive cells decreased to approximately 15%, indicating that SSCs are relatively resistant to Bu-treatment compared to other subtypes of spermatogonia, including differentiating and differentiated.

## Discussion

This study showed that testicular tissue fragments cultured using the PC method can reproduce spermatogenesis efficiently for over 2 months. This in vitro spermatogenesis was disrupted when Bu was added to the culture medium, reflecting the spermatogenesis impairments that were observed in several in vivo studies^22–24,28,29^.

In addition, we found that the Acr-GFP expression area ratio and change in testis tissue volume during cultivation reflected and corresponded with the state of spermatogenesis—being damaged, devastated, or recovering—not only from histological evaluation. Therefore, TOCS with the PC method could be a new system for detecting substances toxic to spermatogenesis.

It was reported recently that the PC method could serve as a potential new method for detecting the toxicity of an anti-cancer drug^33^ and a chemically disturbing hormonal milieu^27^. The present study successfully demonstrated that spermatogenesis impairment can be detected faithfully and evaluated quantitatively using Bu. First, we demonstrated that the concentration of Bu corresponded quite well with the degree of impairment. In toxicological studies, it is crucial to understand the relationship between the dose of a substance being tested and the toxic response exposed subjects demonstrate. In our study, such dose-dependency was evident in the Acr-GFP expression area ratio in each tissue, which was confirmed using histological and IHC evaluations. Furthermore, we have successfully corroborated the results of GFP expression changes and histological evaluation from toxicity onset to recovery by clarifying the behavior of spermatogonia and SSCs, the target cells of Bu toxicity, in organ culture tissues. To the best of our knowledge, this has not been done before.

Meanwhile, a minor difference was recognized in the degree of spermatogenesis impairment between in vivo studies and present TOCS with the PC method. In an in vivo study, a single dose (20 mg/kg) of Bu resulted in an evident impairment of spermatogenesis but was followed by spermatogenic regeneration (Fig. 2a). Exposure of cultured tissues to 40 µM Bu, which was the same concentration of C_max_ measured when 20 mg/kg Bu was administered in vivo, resulted in complete germ cell depletion and no germ cell regeneration was observed. However, we do not regard this kind of discrepancy in the toxic responses as a fundamental difference between in vivo and in vitro systems. The duration of Bu treatment in the cultured tissues was 24 h, whereas plasma Bu was rapidly eliminated from the blood within 8 h after administration (Fig. 2b). In the future, it would be possible to shorten the exposure duration to adjust the area under the curve of Bu concentration between in vivo and in vitro conditions for more precise comparisons.

The reversibility of toxic damage is another principal factor in determining the severity of toxicity in general^34^. In the present study, we demonstrated the recovery of spermatogenesis, which also appeared in the Bu-concentration-dependent manner. In rodents, it takes more than a month from the proliferation of SSCs to the formation of spermatozoa^35–37^; hence, it is necessary to maintain spermatogenesis reliably for such an extended period. Our PC method successfully performed such stable in vitro spermatogenesis for over 10 weeks (Fig. 1c), which is longer than the data in a previous report^21^. These results maintain that the PC method is useful for evaluating the potential of spermatogenic recovery after toxic insults, which is another important aspect of toxicity testing.

In the present study, SALL4-positive cells, i.e., spermatogonia, significantly decreased or disappeared in 1 week of treatment, whereas TRA98-positive cells, i.e., all germ cells, remained in many tubules. This result indicated that Bu toxicity strongly affects spermatogonia among all germ cells, consistent with the previous results^22,23^. In those in vivo studies, germ cells were completely depleted and did not reappear due to the loss of SSCs at high dose Bu exposure. In the 40 μM Bu group of the present study, germ cell depletion was observed in tissues under PC method culture, and only Sertoli cells were observed in the epithelium (Fig. 4b), reproducing a toxic phenotype similar to that observed in in vivo studies. It is well-known that many anti-cancer drugs are toxic to spermatogonia, particularly to differentiated spermatogonia, while undifferentiated spermatogonia, including SSCs, were resistant to them^22,38^. The sensitivity of these two cell populations, differentiated spermatogonia and SSCs, to different drugs was evaluated in mice using the ratio of two LD50 values (SSCs/differentiated spermatogonia)^39^. Among the cytotoxic agents examined, Bu was found to be the most toxic to SSCs compared to differentiated spermatogonia, as indicated by the lowest LD50 ratio of 1.4. In contrast, the ratios for cyclophosphamide and cisplatin were over 7 and 9, respectively^22,38^. The data obtained in the current study may not be adequate for calculating the lethal concentration 50% (LC50), which corresponds to the LD50 in animal experiments, for Bu in SALL4-positive cells (spermatogonia) and GFRA1-positive cells (SSCs). However, it might be possible if several different concentrations of Bu were used in the experiments. Indeed, in this study, the reduction in the numbers of GFRA1-positive cells and SALL4-positive cells in the group treated with 10 µM Bu at the first week of treatment was approximately 70% and 15% of the vehicle group, respectively (Figs. 5d and 6b). These in vitro data have shown that Bu is significantly more cytotoxic to spermatogonia overall than to SSCs, exhibiting approximately 4.7-fold (70%/15%) higher toxicity. Therefore, it is demonstrated that TOCS can replace animal experiments to obtain data such as the LD50 ratio for different cell populations with particular marker proteins identifiable using IHC. In the future, conducting treatments with various low concentrations of Bu is expected to yield more accurate LC50 values, allowing for a more precise comparison of the behavior of germ cells between in vivo studies and organ culture systems.

Changes in testis volume and weight are useful parameters for evaluating testicular damage and impairment of spermatogenesis^40–42^. However, the same was not true in culture experiments in which cultured tissue volume or weight had been rarely measured as an important parameter. In reality, it is not easy to measure accurately the volume or weight of a tiny fragment of testis tissues under cultivation. In this regard, the PC method was revolutionizing, which makes it possible to estimate the volume of cultured tissue even sequentially while keeping the culture experiment continuing by measuring their horizontally projected area^20,27^. It can detect the growth of immature testis tissue over time^20^ and shrinkage when a hormonal chemical is applied^27^. In the present study, the horizontal projected area of cultured tissue decreased in a concentration-dependent manner during the process of impaired spermatogenesis under Bu treatment. This change in tissue volume was supported for the first time by the behavior of germ cells, the toxic target of Bu, which showed a decrease and disappearance over time. These results validate the usefulness of the PC method to provide additional information on tissue volume changes.

It has been proposed that at least 60% of the seminiferous tubules must exhibit spermatogenesis (with spermatocytes and/or spermatids present within them) if we use a TOCS to evaluate testicular toxicity with it^14^. Compared to the AG method, the PC method offers an advantage in maintaining almost the entire tissue in a healthier condition^21^. Consequently, in the present study, more than 60% of the seminiferous tubules cultured with the PC method were spermatogenic, as shown in Supplementary Fig. S2. Thus, the PC method would be more suitable for providing an accurate evaluation of the toxicity of agents of interest.

Ethical considerations regarding animal testing have increased in recent years^43,44^. In line with this thought, it has been reported that TOCS could reduce animal experimentation^10–13^. Specifically, TOCS allows experiments to divide collected testes into multiple tissue pieces. Therefore, it can evaluate each specimen as a single unit instead of each individual animal, in which, unlike with individual animals, exposure conditions can be set separately to each specimen arbitrarily, and each single tissue can be observed sequentially over an extended period, thus reducing the number of experimental animals used.

Although we argued that the PC method has advantages over other TOCSs, the current efficiency of in vitro spermatogenesis is far below that of in vivo spermatogenesis. This is certainly an issue that must be addressed for better performance. Nonetheless, the PC method is useful for efficiently detecting impaired spermatogenesis. The usefulness of the PC method will further increase if it can detect dysfunction of Sertoli cells or Leydig cells caused by toxic substances in the future.

## Methods

### PC chip

PC chips were manufactured and purchased from Fukoku Bussan Co., Ltd. (Tokyo, Japan). The PC chips molded with liquid silicone rubber (ELASTOSIL LR7665 J, Wacker Asahi Kasei Silicone Co., Ltd.) were manufactured using transfer molding. The multi cavity molds for the chips were fabricated using conventional photolithography techniques^45,46^. The molded PDMS piece was cut into a round shape with a diameter of 12 mm. The chip had a circular dent with a diameter of 8 mm, and the dent depth was set to 160 µm, according to a previous report^21^. The PC chips were autoclaved prior to use.

### Animals

Acro3-EGFP B6 transgenic mice^25,26^ were used as the testis tissue source. The Acro3-EGFP B6 transgenic homozygous mouse strain (RBRC00886) was provided by RIKEN BRC through the National BioResource Project of MEXT/AMED, Japan. Wild type C57BL/6N mice (CLEA, Japan) were used for initial breeding and mice carrying transgene were selected for subsequent breeding. Homozygous males of 5–7 dpp were used for the culture experiments. C57BL/6 mice were purchased from Charles River Laboratories Japan, Inc. (Kanagawa, Japan). Mice were maintained at Kao Corporation facility at 22 °C ± 1 °C room temperature, 40–60% humidity, on a 12 h light–dark cycle (7 a.m. to 7 p.m.), and given food and water ad libitum, according to institutional guidelines. All experiments were performed under inhalation anesthesia with isoflurane (Pfizer Inc., USA), and mice were euthanized by cervical dislocation at the end of the experiment. All animal experiments in this study were approved by the Animal Care Committee of Kao Corporation and performed in accordance with the Committee's Guidelines for the Care and Use of Laboratory Animals. We conducted all animal experiments in accordance with ARRIVE guidelines.

### In vivo histological examinations

C57BL/6mice (male, 8 weeks old) were administered intraperitoneal injections of vehicle (dimethylsulfoxide, DMSO), Bu at a dose of 20 mg/kg, or two injections of 20 mg/kg at a 3-h interval as previously reported^28^. Plasma was collected at predetermined intervals over time following Bu injection: for a single-dose injection, samples were collected at 0, 1, 2, 4, and 8 h, and for a two-dose injection, samples were collected at 3, 4, 5, 7, and 11 h. Testes were collected 1, 3, and 5 weeks after treatment and fixed in Bouin's fixative. They were embedded in paraffin blocks, cut into 6-μm-thick sections, and stained with hematoxylin and eosin.

### Quantitative analysis of plasma concentration of Bu

As previously reported, plasma Bu concentration was quantified using liquid chromatography-tandem mass spectrometry (LC–MS/MS)^47^. Solid-phase extraction was performed using an Oasis HLB column (1 cc, 10 mg; Oasis, Milford, MA, USA) to isolate Bu. The plasma samples were diluted tenfold in phosphate-buffered saline (PBS). The HLB column was activated with 1 mL of 90% MeOH containing 2 mM ammonium acetate and 1 mL of water (1 min, vacuum suction). After loading 100 μL of sample onto the column, 1 mL of water and 5% MeOH were used to wash the column. Bu was eluted with 200 μL of 90% MeOH containing 2 mM ammonium acetate and 100 ng/mL busulfan-d8 (230936, Cayman Chemical) as an internal standard (IS). The plasma concentration of Bu was analyzed using an Exion L CAD system coupled with a Triple Quad 5500 + (SCIEX, Framingham, MA, USA). Separations were performed using Acquity UPLC BEH C18 (1.7 μm, 2.1 mm × 50 mm, Waters), maintained at 40 ℃ in a column oven. The mobile phase was 90% MeOH containing 2 mM ammonium acetate buffer, and the flow rate was set to 0.3 mL/min.

MS/MS was equipped with an electrospray ionization source operating in positive ion mode. Quantification was performed in the multiple reaction monitoring (MRM) mode with a mass-to-charge (m/z) transition at 263.9 > 151.0 for Bu and 272.0 > 159.1 for IS. The ionspray voltage and the source temperature were set at 4100 V and 600 ℃, respectively, and the other conditions were set as follows: declustering potential, 26 V for Bu and 56 V for IS; collision cell exit potential, 18 V for Bu and 12 V for IS; entrance potential, 10 V; and collision energy, 17 V. Injections were carried out at 2 μL using an autosampler maintained at 4 ℃.

### PC method and treatment

Testis tissue fragments of 5–7 dpp mice were cultured in α-Minimum essential medium (α-MEM) (12000-022, Gibco), supplemented with 40 mg/mL AlbuMAX (11020-021, Gibco), 2.6% sodium bicarbonate (FUJIFILM Wako, 195-16411), Antibiotic–Antimycotic (15240-062, Gibco), as previously reported^7,21^. The medium was sterilized using a filter with a pore size of 0.10 μm (566-0010, Nalgene) and stored at 4 °C before use. To prepare agarose gel blocks for culture, agarose powder (A426, Dojindo) was dissolved in distilled water to 1.5% (wt/vol) and autoclaved. The agarose solution (33 mL) was poured into 10 cm dishes to form an approximately 5 mm thick gel. The gel was cut into approximately 1.5 cm^2^ pieces which were used as stands for the placement of the testis tissue fragments. Before culturing, the gel blocks were submerged in the culture medium for 2 days, which was changed daily. The decapsulated testes were divided into approximately 6–8 pieces using forceps. The tissue fragments were placed on an agarose gel block half-soaked in a culture medium in a 6-well culture plate (3513, Corning). Subsequently, the PC chip was placed over the testis fragment, dent‐side down. The medium was added to 2 mL of each well, and the solution was changed once a week. The culture incubator was supplied with 5% CO_2_ in air and maintained at 34 °C.

Cultured testis tissue fragments were treated with 10, 20, and 40 µM Bu (B2635-10G, Sigma-Aldrich). The same concentration of DMSO was used as a vehicle control. After 24 h of treatment, the medium was removed, and the cells were washed thrice (every 3 h) with fresh medium, followed by observation under an inverted microscope. The cultured tissues were harvested for histochemical evaluation at each time period (1, 3, and 5 weeks).

### Observation of cultured tissues

Cultured tissues were observed under a stereomicroscope (LeicaM205 FA; Leica) over time to capture GFP expression and bright-field images during the culture. The cultured tissues were observed using an inverted fluorescence microscope (BZ-X700, KEYENCE, Osaka, Japan) to identify haploid cells with an acrosome cap structure.

### Histological and IHC examinations

For histological examinations, specimens were fixed with Bouin's fixative, embedded in paraffin, and cut into 6-μm thick sections horizontally to achieve the largest cross-sectional area. Each specimen was stained with hematoxylin and eosin (H&E) or PAS.

For IHC examination of paraffin-embedded sections, specimens were fixed with 10% formalin neutral buffer solution (133-10311, FUJIFILM Wako Pure Chemical Corporation) at 4 °C overnight. They were embedded in paraffin blocks and cut into 7-μm-thick horizontal sections. Dewaxed and rehydrated sections were treated with antigen retrieval in citrate buffer (pH 6.0) (S2369, DAKO) at 98 ℃ for 10 min, and 10% normal goat serum (426042; Nichirei Biosciences Inc.) was used for blocking nonspecific binding for 1 h at room temperature or used as an antibody dilution. Sections were incubated with primary antibodies overnight at 4 ℃. After several washes, sections were incubated with secondary antibodies diluted in PBS containing 4ʹ,6-diamidino-2-phenylindole (DAPI) (D523; Dojindo, Kumamoto, Japan) for 1 h at room temperature in the dark. The sections were washed several times with PBS and mounted on raised coverslips using mounting medium (0100-01, Southern Biotechnology Associates, Inc.).

The primary antibodies used for IHC (paraffin sections) included anti-TRA98 (1:200, ab82527, abcam), anti-SALL4 (1:200, ab57577, abcam), anti-alpha-smooth muscle actin (α-SMA)-Alexa Fluor647 (1:200, ab202296, abcam), and anti-WT1 (1:200, mab4234, Millipore). The secondary antibodies used were Alexa Fluor 555-conjugated goat anti-rat antibody (1:500, A-48263, Invitrogen) and Alexa Fluor 555-conjugated goat anti-mouse antibody (1:500, A-21127, Invitrogen). Observations of immunostained sections were performed with an inverted fluorescence microscope (BZ700, Keyence, Japan).

For IHC examinations for frozen sections (GFRα1; GFP detection), the specimens were fixed with 4% paraformaldehyde in PBS overnight at 4 °C. Fixed tissues were soaked in 30% (w/v) sucrose in PBS at 4 °C overnight. They were cryo-embedded in OCT compound (Sakura Finetek Japan) and cut into 6-μm-thick sections horizontally. The cryosections were washed thrice with TBS (Bio-Rad Laboratories, Inc., 1706435) for 5 min.

To detect the GFRα1 signal, 4% normal donkey serum (HR-8135, ImmunoBioScience Corp.) containing 0.5% blocking reagent (PF1020, AKOYA Biosciences) in TBS was used as the blocking solution or for primary and secondary antibody dilutions. TBS-T (0.1% Tween 20 in TBS) was used as the washing solution. The primary antibody used was anti-GFRα1 (1:200, AF560, Bio-Techne). Sections were blocked for 1 h at room temperature and incubated with primary antibodies overnight at 4 ℃. The secondary antibody was donkey anti-goat IgG conjugated to Alexa Fluor 555 (1:500, A32816, Invitrogen). Cells were incubated with DAPI for 1 h at room temperature in the dark. The sections were washed several times with PBS and mounted as previously described.

To detect the GFP signal, 10% normal goat serum was used as the blocking solution or for primary and secondary antibody dilutions. Sections were blocked for 1 h at room temperature and incubated with primary antibodies overnight at 4 ℃. The primary antibody was anti-GFP (1:400, 632569; Takara Bio Inc.). Lectin peanut agglutinin (PNA) from *Arachis hypogaea* (peanut) and Alexa Fluor 647 conjugate (1:500, L32458, Invitrogen) were used to identify acrosomes. The secondary antibody, Alexa Fluor 555-conjugated goat anti-mouse antibody (1:500, A21127, Invitrogen), was incubated with DAPI for 1 h at room temperature in the dark. The sections were washed several times with PBS and mounted as previously described. The immunostained sections were observed using an inverted fluorescence microscope (BZ-X700; KEYENCE, Osaka, Japan).

### Quantitative image analysis

The horizontal projection area of the cultured tissue and GFP-expression area ratio were calculated from six cultured tissues at each concentration using a hybrid cell count application (BZ-H4C, KEYENCE, Osaka, Japan) in BZ-X Analyzer software (BZ-H4A, KEYENCE, Osaka, Japan). For IHC, 4 cultured tissues from each Bu treatment group were used for quantitative analysis. Four representative areas of tissue sections at each concentration were used to quantify the IHC images. Positive areas were calculated using a hybrid cell count application as described above.

### Statistical analysis

Each data point, or bar graph, represents the mean ± SD. Statistical analyses were performed using GraphPad Prism (version 9) statistical software (GraphPad Software, San Diego, CA). Statistical analyses were performed using one-way ANOVA followed by the Tukey–Kramer Test. *P* < 0.05 were considered to indicate statistical significance.

## Supplementary Information

Supplementary Figures.

## Data Availability

All data analyzed in this study are included in this published article and the Supplementary Information file.

## References

[1] Cox, C. M. *et al.* Infertility prevalence and the methods of estimation from 1990 to 2021: A systematic review and meta-analysis. *Hum. Reprod. Open* **2022**, hoac051. 10.1093/hropen/hoac051 (2022). 36483694 10.1093/hropen/hoac051 PMC9725182

[2] Leslie, S. W., Soon-Sutton, T. L. & Khan, M. A. B. Male Infertility. *StatPearls* (StatPearls Publishing Copyright © 2023, StatPearls Publishing LLC., 2023).

[3] Agarwal, A. *et al.* Male infertility. *Lancet* **397**, 319–333. 10.1016/s0140-6736(20)32667-2 (2021). 33308486 10.1016/s0140-6736(20)32667-2

[4] Yoshida, S. Mouse spermatogenesis reflects the unity and diversity of tissue stem cell niche systems. *Cold Spring Harb. Perspect. Biol.*10.1101/cshperspect.a036186 (2020). 32152184 10.1101/cshperspect.a036186 PMC7706566

[5] Trost, N., Mbengue, N. & Kaessmann, H. The molecular evolution of mammalian spermatogenesis. *Cells Dev.* **175**, 203865. 10.1016/j.cdev.2023.203865 (2023). 37336426 10.1016/j.cdev.2023.203865 PMC10363733

[6] Mäkelä, J. A., Koskenniemi, J. J., Virtanen, H. E. & Toppari, J. Testis development. *Endocr. Rev.* **40**, 857–905. 10.1210/er.2018-00140 (2019). 30590466 10.1210/er.2018-00140

[7] Sato, T. *et al.* In vitro production of functional sperm in cultured neonatal mouse testes. *Nature* **471**, 504–507. 10.1038/nature09850 (2011). 21430778 10.1038/nature09850

[8] Sanjo, H. *et al.* Antioxidant vitamins and lysophospholipids are critical for inducing mouse spermatogenesis under organ culture conditions. *FASEB J.* **34**, 9480–9497. 10.1096/fj.202000245R (2020). 32474967 10.1096/fj.202000245R

[9] Matsumura, T. *et al.* Rat in vitro spermatogenesis promoted by chemical supplementations and oxygen-tension control. *Sci. Rep.* **11**, 3458. 10.1038/s41598-021-82792-2 (2021). 33568686 10.1038/s41598-021-82792-2 PMC7875995

[10] Brannen, K. C., Chapin, R. E., Jacobs, A. C. & Green, M. L. Alternative models of developmental and reproductive toxicity in pharmaceutical risk assessment and the 3Rs. *ILAR J.* **57**, 144–156. 10.1093/ilar/ilw026 (2016). 28053068 10.1093/ilar/ilw026

[11] Nakamura, N., Sloper, D. T. & Del Valle, P. L. Evaluation of an in vitro mouse testis organ culture system for assessing male reproductive toxicity. *Birth Defects Res.* **111**, 70–77. 10.1002/bdr2.1431 (2019). 30575315 10.1002/bdr2.1431

[12] Park, H. J., Lee, W. Y., Do, J. T., Park, C. & Song, H. Evaluation of testicular toxicity upon fetal exposure to bisphenol A using an organ culture method. *Chemosphere* **270**, 129445. 10.1016/j.chemosphere.2020.129445 (2021). 33421752 10.1016/j.chemosphere.2020.129445

[13] Park, H. J., Kim, J. S., Lee, R. & Song, H. Cisplatin induces apoptosis in mouse neonatal testes organ culture. *Int. J. Mol. Sci.*10.3390/ijms232113360 (2022). 36362147 10.3390/ijms232113360 PMC9658841

[14] Chapin, R. E. *et al.* Lost in translation: The search for an in vitro screen for spermatogenic toxicity. *Birth Defects Res. B Dev. Reprod. Toxicol.* **107**, 225–242. 10.1002/bdrb.21188 (2016). 28024311 10.1002/bdrb.21188

[15] Komeya, M. *et al.* Long-term ex vivo maintenance of testis tissues producing fertile sperm in a microfluidic device. *Sci. Rep.* **6**, 21472. 10.1038/srep21472 (2016). 26892171 10.1038/srep21472 PMC4759809

[16] Komeya, M. *et al.* Pumpless microfluidic system driven by hydrostatic pressure induces and maintains mouse spermatogenesis in vitro. *Sci. Rep.* **7**, 15459. 10.1038/s41598-017-15799-3 (2017). 29133858 10.1038/s41598-017-15799-3 PMC5684205

[17] Yamanaka, H. *et al.* A monolayer microfluidic device supporting mouse spermatogenesis with improved visibility. *Biochem. Biophys. Res. Commun.* **500**, 885–891. 10.1016/j.bbrc.2018.04.180 (2018). 29705697 10.1016/j.bbrc.2018.04.180

[18] AbuMadighem, A., Shuchat, S., Lunenfeld, E., Yossifon, G. & Huleihel, M. Testis on a chip-a microfluidic three-dimensional culture system for the development of spermatogenesisin-vitro. *Biofabrication.*10.1088/1758-5090/ac6126 (2022). 35334473 10.1088/1758-5090/ac6126

[19] Önen, S. *et al.* A pumpless monolayer microfluidic device based on mesenchymal stem cell-conditioned medium promotes neonatal mouse in vitro spermatogenesis. *Stem Cell Res. Ther.* **14**, 127. 10.1186/s13287-023-03356-x (2023). 37170113 10.1186/s13287-023-03356-x PMC10173473

[20] Kojima, K. *et al.* Neonatal testis growth recreated in vitro by two-dimensional organ spreading. *Biotechnol. Bioeng.* **115**, 3030–3041. 10.1002/bit.26822 (2018). 30144353 10.1002/bit.26822 PMC6283240

[21] Komeya, M. *et al.* In vitro spermatogenesis in two-dimensionally spread mouse testis tissues. *Reprod. Med. Biol.* **18**, 362–369. 10.1002/rmb2.12291 (2019). 31607796 10.1002/rmb2.12291 PMC6780044

[22] Bucci, L. R. & Meistrich, M. L. Effects of busulfan on murine spermatogenesis: Cytotoxicity, sterility, sperm abnormalities, and dominant lethal mutations. *Mutat. Res.* **176**, 259–268. 10.1016/0027-5107(87)90057-1 (1987). 3807936 10.1016/0027-5107(87)90057-1

[23] Chen, X., Liang, M. & Wang, D. Progress on the study of the mechanism of busulfan cytotoxicity. *Cytotechnology* **70**, 497–502. 10.1007/s10616-018-0189-5 (2018). 29350306 10.1007/s10616-018-0189-5 PMC5851972

[24] Choi, Y. J. *et al.* Murine male germ cell apoptosis induced by busulfan treatment correlates with loss of c-kit-expression in a Fas/FasL- and p53-independent manner. *FEBS Lett.* **575**, 41–51. 10.1016/j.febslet.2004.08.034 (2004). 15388331 10.1016/j.febslet.2004.08.034

[25] Nakanishi, T. *et al.* Real-time observation of acrosomal dispersal from mouse sperm using GFP as a marker protein. *FEBS Lett.* **449**, 277–283. 10.1016/s0014-5793(99)00433-0 (1999). 10338148 10.1016/s0014-5793(99)00433-0

[26] Ohta, H., Sakaide, Y. & Wakayama, T. Functional analysis of male mouse haploid germ cells of various differentiation stages: early and late round spermatids are functionally equivalent in producing progeny. *Biol. Reprod.* **80**, 511–517. 10.1095/biolreprod.108.073270 (2009). 19073998 10.1095/biolreprod.108.073270

[27] Hashimoto, K. *et al.* Culture-space control is effective in promoting haploid cell formation and spermiogenesis in vitro in neonatal mice. *Sci. Rep.* **13**, 12354. 10.1038/s41598-023-39323-y (2023). 37524742 10.1038/s41598-023-39323-y PMC10390558

[28] Xie, Y. *et al.* Establishing a nonlethal and efficient mouse model of male gonadotoxicity by intraperitoneal busulfan injection. *Asian J. Androl.* **22**, 184–191. 10.4103/aja.aja_41_19 (2020). 31187778 10.4103/aja.aja_41_19 PMC7155790

[29] Wang, D. Z., Zhou, X. H., Yuan, Y. L. & Zheng, X. M. Optimal dose of busulfan for depleting testicular germ cells of recipient mice before spermatogonial transplantation. *Asian J. Androl.* **12**, 263–270. 10.1038/aja.2009.67 (2010). 20010847 10.1038/aja.2009.67 PMC3739084

[30] Chan, A. L. *et al.* Germline stem cell activity is sustained by SALL4-dependent silencing of distinct tumor suppressor genes. *Stem Cell Rep.* **9**, 956–971. 10.1016/j.stemcr.2017.08.001 (2017). 10.1016/j.stemcr.2017.08.001 PMC5599261 28867346

[31] Buageaw, A. *et al.* GDNF family receptor alpha1 phenotype of spermatogonial stem cells in immature mouse testes. *Biol. Reprod.* **73**, 1011–1016. 10.1095/biolreprod.105.043810 (2005). 16014811 10.1095/biolreprod.105.043810

[32] Lucas, B., Fields, C. & Hofmann, M. C. Signaling pathways in spermatogonial stem cells and their disruption by toxicants. *Birth Defects Res. C Embryo Today* **87**, 35–42. 10.1002/bdrc.20145 (2009). 19306349 10.1002/bdrc.20145 PMC2906709

[33] Hashimoto, K. *et al.* A novel alternative method for long-term evaluation of male reproductive toxicity and its recovery using a pre-pubertal mouse testis organ culture system. *J. Appl. Toxicol.*10.1002/jat.4584 (2024). 38262615 10.1002/jat.4584

[34] International Council for Harmonisation of Technical Requirements for Pharmaceuticals for Human Use. ICH HARMONISED GUIDELINE. Detection of Reproductive and Developmental Toxicity for Human Pharmaceuticals S5(R3). May 2021. ICH Revision 3. Final version, Adopted on 18 February 2020. https://database.ich.org/sites/default/files/S5R3_Step4_Guideline_2020_0218_1.pdf.

[35] Oakberg, E. F. Duration of spermatogenesis in the mouse and timing of stages of the cycle of the seminiferous epithelium. *Am. J. Anat.* **99**, 507–516. 10.1002/aja.1000990307 (1956). 13402729 10.1002/aja.1000990307

[36] Clermont, Y. & Harvey, S. C. Duration of the cycle of the seminiferous epithelium of normal, hypophysectomized and hypophysectomized-hormone treated albino rats. *Endocrinology* **76**, 80–89. 10.1210/endo-76-1-80 (1965). 14254202 10.1210/endo-76-1-80

[37] França, L. R., Ogawa, T., Avarbock, M. R., Brinster, R. L. & Russell, L. D. Germ cell genotype controls cell cycle during spermatogenesis in the rat. *Biol. Reprod.* **59**, 1371–1377. 10.1095/biolreprod59.6.1371 (1998). 9828180 10.1095/biolreprod59.6.1371

[38] Meistrich, M. L. Stage-specific sensitivity of spermatogonia to different chemotherapeutic drugs. *Biomed. Pharmacother.* **38**, 137–142 (1984). 6478053

[39] Meistrich, M. L., Finch, M., da Cunha, M. F., Hacker, U. & Au, W. W. Damaging effects of fourteen chemotherapeutic drugs on mouse testis cells. *Cancer Res.* **42**, 122–131 (1982). 7198505

[40] Christian, M. S. Review of reproductive and developmental toxicity of 1,3-butadiene. *Toxicology* **113**, 137–143. 10.1016/0300-483x(96)03438-5 (1996). 8901893 10.1016/0300-483x(96)03438-5

[41] Zubair, M. *et al.* Protective effects of vitamin E on sodium arsenite-induced toxicity, testicular measurements and histopathological studies of testes in Teddy goat bucks. *Andrologia.*10.1111/and.12699 (2017). 27686236 10.1111/and.12699

[42] Akhigbe, R. & Ajayi, A. Testicular toxicity following chronic codeine administration is via oxidative DNA damage and up-regulation of NO/TNF-α and caspase 3 activities. *PLOS ONE* **15**, e0224052. 10.1371/journal.pone.0224052 (2020). 32168344 10.1371/journal.pone.0224052 PMC7069647

[43] Stokes, W. S. Animals and the 3Rs in toxicology research and testing: the way forward. *Hum. Exp. Toxicol.* **34**, 1297–1303. 10.1177/0960327115598410 (2015). 26614819 10.1177/0960327115598410

[44] Lewis, D. I. Animal experimentation: Implementation and application of the 3 Rs. *Emerg. Top. Life Sci.* **3**, 675–679. 10.1042/etls20190061 (2019). 32915219 10.1042/etls20190061

[45] Duffy, D. C., McDonald, J. C., Schueller, O. J. & Whitesides, G. M. Rapid prototyping of microfluidic systems in poly(dimethylsiloxane). *Anal. Chem.* **70**, 4974–4984. 10.1021/ac980656z (1998). 21644679 10.1021/ac980656z

[46] Fujii, T. PDMS-based microfluidic devices for biomedical applications. *Microelectron. Eng.* **61–62**, 907–914 (2002). 10.1016/S0167-9317(02)00494-X

[47] Matar, K. M., Alshemmari, S. H., Refaat, S. & Anwar, A. UPLC-tandem mass spectrometry for quantification of busulfan in human plasma: Application to therapeutic drug monitoring. *Sci. Rep.* **10**, 8913. 10.1038/s41598-020-65919-9 (2020). 32488110 10.1038/s41598-020-65919-9 PMC7265561

