# Supplementary material for: Application of testicular organ culture system for the evaluation of spermatogenesis impairment
Source: Sci Rep. 2024 Sep 16;14:21581. doi: 10.1038/s41598-024-71561-6 (PMC11405715; doi:10.1038/s41598-024-71561-6)
Supplement: Supplementary file 1 — Supplementary Figures. [file 41598_2024_71561_MOESM1_ESM.pdf]

Supplementary Figure S1.

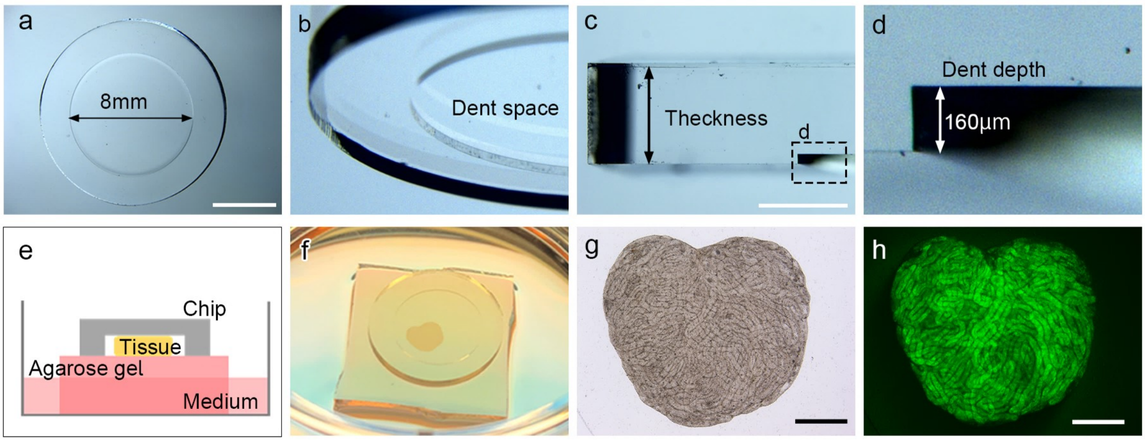

PDMS ceiling chip and method.

(a) The PDMS ceiling chip used in this study was round-shaped, with a diameter of 12 mm. (b) The PC chip panel reveals a central dent space. (c, d) A vertically halved section of the PC chip illustrating thickness and dent depth.The PC chip features a 160 μm dent depth designed to enclose the tissue. (e) Schematics of the PC method. The PC chip is positioned over the testis fragment, with the dented side facing downward. (f-h) On day 33 of cultured tissue observation, GFP expression was observed throughout the entire area of the tissue fragment. Scale bars: 5 mm (a), 1 mm (c), and 2 mm (g, h).

Supplementary Figure S2.

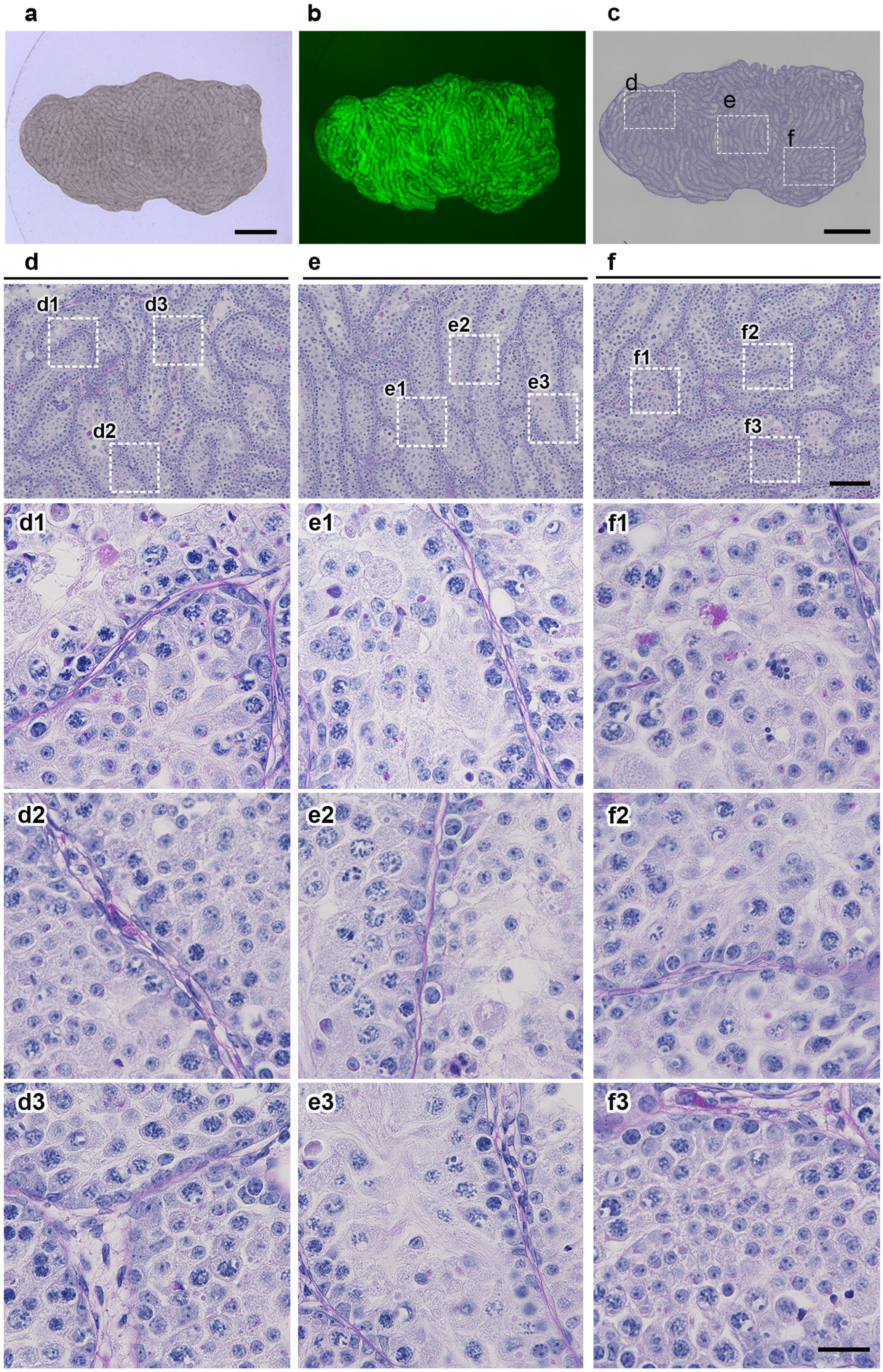

Histological evaluation of testes fragments cultured using the PC method.

(a, b) Stereomicroscopic view of testis tissue from a 6 dpp mouse cultured for 42 days. (a) Bright-field and (b) GFP expression. (c-f) PAS stain of the same specimen. Dashed-line frames are magnified in the panel with corresponding letters. Scale bars: 1 mm (a, b, c), 100 μm (d, e, f), and 20 μm (d1-3, e1-3, f1-3).
